# Measuring dexterity in the podiatrist population: a cross-sectional comparison of novice students and experienced podiatrists

**DOI:** 10.1186/s12909-018-1276-1

**Published:** 2018-08-02

**Authors:** Ryan Causby, Michelle McDonnell, Lloyd Reed, Susan Hillier

**Affiliations:** 1 Sansom Institute for Health Research, University of South Australia, North Terrace, GPO Box 2471, Adelaide, SA 5001 Australia; 2 Alliance for Research in Exercise, Nutrition and Activity, University of South Australia, North Terrace, Adelaide, SA 5000 Australia; 3 School of Clinical Sciences, Queensland University of Technology, Kelvin Grove, Qld Australia

**Keywords:** Dexterity, Podiatry, Psychomotor testing, Dexterity testing

## Abstract

**Background:**

There is no ‘gold-standard’ for the evaluation of dexterity for the health professional or podiatrist populations. This has resulted in a broad array of generalised tests to evaluate dexterity. Thus, the aim was to determine which objective generalised dexterity tests are best suited to evaluating dexterity in a podiatry student population.

**Methods:**

A cohort of *Novice* podiatry students and *Experienced* podiatrists were recruited and evaluated on a battery of dexterity tests selected to evaluate a variety of different elements. Group differences were evaluated statistically and regression undertaken on significant test outcomes.

**Results:**

A total of 108 participants were recruited with 54 participants in each of the Novice and Experienced groups. Five of the eight tests were able to discriminate dexterous ability of participants in the *Novice* and *Experienced* groups. These included the Grip-lift task, GPT, P-MVC, G-MVC and the AsTex® sensory discrimination test. These tests comprised a total of 11 significant dependent variables (*p* <  0.05). From the test battery, outcomes were able to predict 79% of the group membership. Age and experience did not explain within-group variability for the *Experienced* group.

**Conclusion:**

Whilst the *Experienced* group displayed superior performance in strength and speed, the *Novice* group showed superior coordination and sensory ability. From these findings, we would recommend that outcomes from the Grooved Pegboard Test, Grip-lift task, Grip Strength test and Pinch Grip strength test be used to evaluate elements of dexterity in this population.

**Electronic supplementary material:**

The online version of this article (10.1186/s12909-018-1276-1) contains supplementary material, which is available to authorized users.

## Background

The ability to perform fine and gross motor activities, in addition to a sound theoretical knowledge, is an important component of health professional practice. A good example of this is the podiatric profession.

Numerous methods of evaluating dexterity have been proposed and used for a wide variety of purposes, but only a limited number of these tests have been used to evaluate a health profession population [1] and only one study has addressed the evaluation of dexterity in a podiatrist population [2]. To date there is no ‘gold-standard’ for the evaluation of dexterity, either generally for health professionals or podiatrists specifically.

One reason for the lack of a gold-standard for measuring dexterity may be the number of psychomotor elements which contribute to a dexterous performance. For example, when using a scalpel, in addition to the co-ordination of muscle groups to effectively and appropriately facilitate scalpel movement, numerous afferent and cognitive processes are required. These include visualisation, proprioceptive and tactile sensation; predictive processes determine the appropriate amount of force and approach required and cognitive processes to integrate and interpret this input in order to successfully coordinate and implement a suitable motor plan. Task requirements in all of these foundational areas can vary from slow, precise, fine movements in a surgical nail bed resection through to the rapid, more forceful, repetitive movements required for callus debridement. Tests of dexterity target different components which contribute to a dexterous performance and consequently may suit one particular task (or profession) better than another.

Thus, the primary aim of this study was to determine which objective generalized dexterity tests (GDTs) are best suited to evaluating dexterity in those with and without experience as a podiatrist using scalpels. The assumption being that an experienced podiatrist would have better dexterity and thus perform better. Tests were selected which targeted different elements of dexterity. The intended outcomes were two-fold; firstly, to determine which tests were able to discriminate between the novice students and experienced podiatrists and secondly, to determine which of these contributed the most to group classification, allowing us to identify which elements may be most relevant to podiatric experience and provide further insight into the evaluation of dexterity in the health professions. In order to consider these aims the following questions were contemplated: Which dependent test outcomes significantly differentiate the experience between groups? Which outcome measures best categorize participants appropriately into experience groups? Were there any within-group confounders for test variables based on actual level of experience in the experienced group?

## Methods

A cohort of podiatry students were recruited from the University of South Australia (UniSA) and the Queensland University of Technology (QUT) (Novice group), with ethics approval from both sites and consent sought in line with the Declaration of Helsinki. Experienced group participants were recruited from the podiatry workforce. Participants were required to be between 18 and 40 years of age, not perform as a professional musician, or have a condition or require medication which could affect hand function. Specifically professional musicians were excluded due to evidence for occupation-related improvements in haptic sense [3]. Novice participants were first or second year students who had not used a scalpel previously. Experienced participants needed to have greater than 2 years of podiatric clinical experience working greater than 0.5 full-time equivalent. The 2 year period was selected as an arbitrary figure to ensure that participants had extensive experience whilst minimizing the chances of having an experienced group which was significantly older than the novice group as some of the tests may be sensitive to aged-related changes in performance [1].

Demographic information, including height, age, sex, medical history, working history, hobbies including current or previous computer game or musical instrument playing, and podiatric experience were recorded for each participant. Participants who identified as having played computer games or musical instruments as a hobby were required to provide an estimate of time dedicated to the task in order to further explore the effects on test variables if necessary. This was achieved through the selection of a grouped category, being: ‘nil’, ‘less than five hours’, ‘between five and ten hours’ and ‘greater than ten hours’ per week. Participants from the Experienced group also made an estimation of the approximate number of patients seen to date to differentiate experience levels if further within-group discrimination was required. Each participant’s hand dominance was determined using the Edinburgh Handedness Inventory (EHI) [4].

Participants were required to perform a battery of objective psychomotor tests in a single 60 min session (Table 1). Participants were requested not to consume caffeine or other stimulants, or undertake any vigorous exercise on the day of testing. Testing was undertaken with participants sitting at an appropriate height to avoid any influence from working in a compromised position [5]. The dominant hand was tested first, followed by the non-dominant hand. In participants who were identified as ambidextrous by the EHI (laterality quotient between − 40 and + 40), the writing-hand was tested first.

**Table 1:** Outcome variables for each of the tests within the battery

Test (in order of testing)	Outcome Variables
Tremor	Peak power in acceleration spectrum (g^2^), Frequency of peak power (Hz)
Visuomotor tracking test (VTT)	Absolute error value (deg), Maximum cross-correlation value (ρ), lag (ms)
Finger Tapping Test (FTT)	Maximum taps (n), Coefficient of variation
Maximum Pinch Grip (P-MVC)	Maximum force (N)
Grip-Lift Task	Preload duration (ms), Minimum Load (N), Maximum grip force (N), Grip force to Lift force ratio, Maximum cross-correlation (ρ), Timeshift (ms), Average Grip (N), Standard Deviation of grip, Hold ratio, Lift Duration (ms)
Grooved Pegboard Test (GPT)	Time to complete (s)
Grip Strength (G-MVC)	Maximum Force (Kgs)
AsTEX ® Sensory test	Texture Discrimination Index (mm)

### Dexterity tests

The test battery is presented in order of testing in Table 1.

#### Tremor

Tremor was measured and analysed using methods similar to that of Flavel et al. [6]. Resting tremor was measured with the hand resting on the table in a self-selected comfortable position. Tremor during an externally paced (metronome at 1 Hz) flexion and extension movement of the index finger was then performed with the participant’s hand off the table in front of them in a comfortable position with the arm unsupported, shoulder in a neutral position, the elbow flexed and forearm pronated. A minimum period of 30 s of data was recorded for both conditions. As physiological tremor occurs between 8 and 12 Hz in healthy people [7], the peak power and frequency within a 7–13 Hz range were extracted. The mean power for the same range was also determined for comparison between trials. Mean and peak power are an index of tremor amplitude in the specified frequency range [7].

#### Visuomotor tracking task (VTT)

**Fig. 1:**
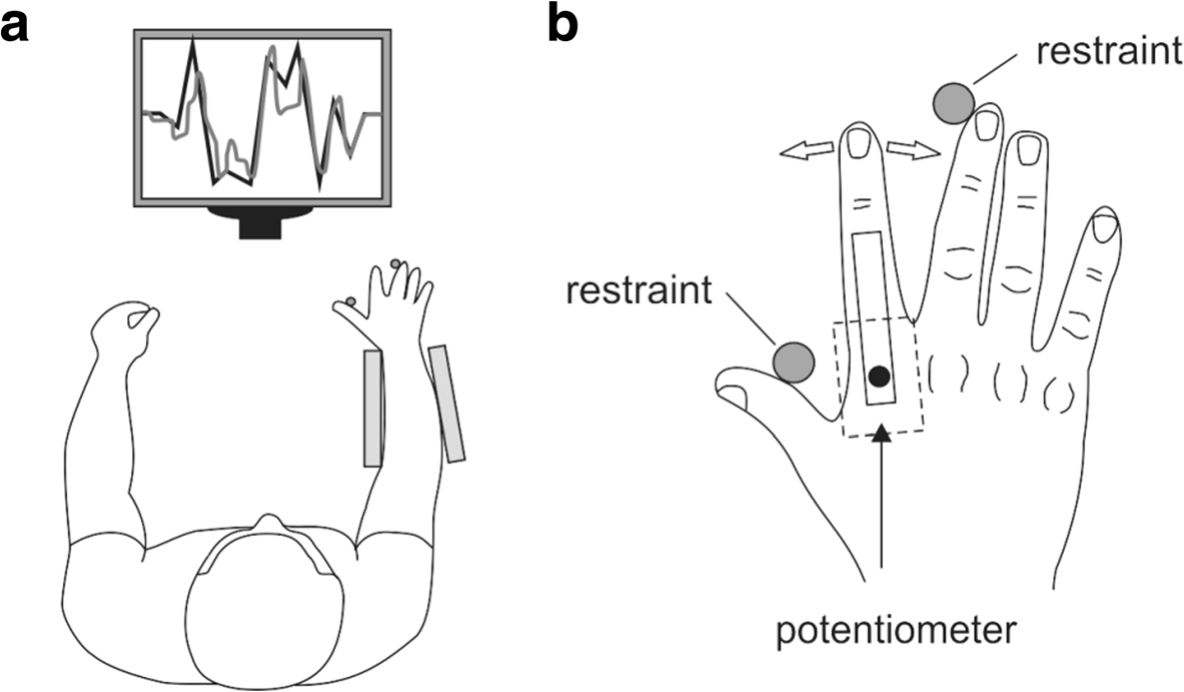
Visuomotor tracking task (VTT) apparatus (adapted from [40], p 3). **a** Overview of VTT apparatus setup with participant sitting at a desk. **b** Close-up of electrogoniometer setup on the dominant hand

The VTT used a device and methods similar to that described by Todd et al. [8]. The VTT requires participants to manipulate a waveform graph on a computer screen via an electrogoniometer attached to the index finger to match a target wave (Fig. 1). In order to manipulate the cursor, the index finger must be abducted and adducted at the metacarpophalangeal joint which facilitates onscreen movement of the cursor to the top or bottom of the screen. The target path is created by 18 individual epochs of 10 s duration, in which the line moves from horizontal up and down up to the equivalent of ±10 degrees on the goniometer in an unpredictable pattern (Fig. 1). Participants were instructed to follow the path as closely as possible, moving the finger when the target path moved from being a horizontal line. An acclimatisation period was not provided. Data analysis, as outlined in Todd et al. [8], involved the extraction of target and goniometer data points to Microsoft Excel™ (Microsoft Corporation, 2010) so that the absolute error (the distance between the target and tracking path at each time point) could be calculated; the average for each epoch was calculated. The remaining outcomes required the transfer of each specified time period to SPSS v.21 (IBM Corporation, Armonk, NY, USA) for a cross-correlation analysis of target versus tracking values. Cross-correlation was set to ±200 lags (ms); the maximum cross-correlation value and lag point at which this occurred were exported for statistical group comparisons.

#### Grip-lift task

**Fig. 2:**
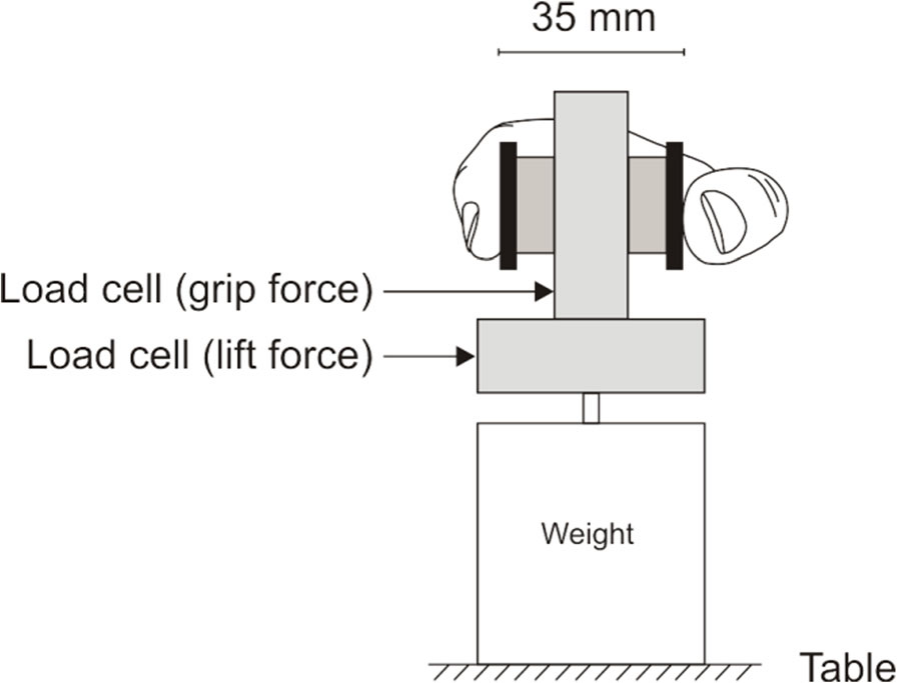
Grip Lift Manipulandum (adapted from [41], p 5)

The Grip-Lift task uses a manipulandum similar to that used by Westling and Johansson [9] (Fig. 2). The manipulandum is a device for measuring grip force and lift force whilst a small object is gripped and lifted off the supporting surface. Two linear strain gauges (model MLP-100; Transducer Techniques, Temecula, CA, USA) detect the grip force (horizontal force) applied to the object whilst simultaneously recording lift forces (vertical) as the device is lifted to a pre-determined height (100 mm). Two brass pads are positioned at the top of the device and sit approximately 35 mm apart. Below the second force gauge there is a metal strip which provides a ledge upon which a variety of weights may be placed to alter the overall weight of the device. This weight is indeterminable to the participant to prevent visual based prediction of weight whereby they could establish an anticipatory strategy for lifting the manipulandum. To lift the device, participants used a pincer-style grip also known as a precision grip [9–11], similar to the thumb and forefinger grip commonly used to hold a scalpel handle. Participants were required to wash their hands to remove sweat and oily substances as required for the Grip-Lift task [9, 12]. Methods employed were similar to those reported by McDonnell et al. [13] and Todd et al. [8]. Briefly, the participant was required to gently lift (lift phase), hold (hold phase) and replace the manipulandum on the table. This process was repeated for three trials on each hand with a rest period of approximately 10 s between trials to allow adequate data separation for analysis. The outcomes of interest are: Preload duration (PDn), Minimum Load (LFmin), Maximum grip force (GFmax), Grip force to Lift force ration (GF:LF), Maximal cross-correlation, Timeshift, Lift duration (LFDn), Average grip force (GFavg), Standard deviation of grip force (GFsd), Hold Ratio. Further details of these outcomes can be found in Additional file 1.

For analysis, each of the files were printed and visually inspected for any notable fumbled or failed attempts, which were then removed from further analysis. Although a fumble can be indicative in itself of poor technique or strategy, the procedure used to analyze the data would result in the reporting of false values for some of the variables. A total of 19 lifts for the dominant hand (11 Experienced and 8 Novice) and 14 lifts for the non-dominant hand (6 Experienced and 8 Novice) were removed However, as a consequence of the removal of a number of first lifts due to fumbles and the potential skewing of the data, whereby data would be removed for the worst performers only, it was decided to use the average of the second and third lifts for analysis. This has the potential benefit of also providing a better representation of a participant’s learning over the repetitive lifts, without the skewing an initial poor performance during the early error-based (fast) learning.

The following operational definitions based on the methods of McDonnell et al. [11] and Duque et al. [14] were used for analysis: Pre-lift phase, grip onset, Lift onset, Lift phase and Hold phase (see Additional file 1 for further information).

#### Finger tapping test (FTT)

The FTT [15] is a measure of motor speed and coordination control [16]. The method of administration used was similar to Todd et al. [8], whereby participants were required to tap the linear strain gauge associated with the grip lift manipulandum (Fig. 2) for 10s as it lay on its side. The heel of the hand was required to maintain contact with the bench top to avoid participants using their whole arm. The following outcomes were analysed: number of taps, inter-tap interval and coefficient of variation (for the taps) variables.

#### Pinch grip strength – Pinch maximum voluntary contraction (P-MVC)

The Pinch Grip, or Pinch Maximum Voluntary Contraction (P-MVC) test may also be termed the tip pinch [17] or strength of the precision grip [11]. P-MVC involves gripping the manipulandum, with the hand supinated, between the tips of the thumb and index finger without recruiting adjacent digits. Three trials were performed with a 30 s rest period in between trials.

#### Grooved pegboard test (GPT)

The GPT [18] (model 32,025, Lafayette Instrument, Lafayette, IN, USA) has 25 keyhole style slots of varying orientation throughout. Pins of key-like presentation to be aligned to the holes and inserted accurately. The GPT is purported to test psychomotor speed, fine motor control, and rapid visual-motor coordination [16]. It is likely that greater sensory feedback is required to sense the orientation of the groove on the pin prior to placement and that greater coordination is required than the Purdue Pegboard [19]. The standard testing protocol reported by Trites [18] was followed. Three trials were undertaken for each hand, with the fastest time taken to complete the board recorded for analysis. A 30 s rest period was provided between trials to allow the pegboard to be re-set and prevent participant fatigue.

#### Grip strength – Grip maximum voluntary contraction (G-MVC)

The Grip Strength, or Grip Maximum Voluntary Contraction (G-MVC) test is a measure of efferent output, which was captured with a hand-held dynamometer (Jamar Dynamometer - Sammons Preston Rolyan, Bolingbrook, IL, USA). To perform this test a dynamometer was held by the participant with the shoulder in adduction and neutral humeral rotation, the elbow flexed to 90° and the forearm and wrist in mid-pronation [20]. Participants then squeezed the handgrip as hard as possible in a rapid, maximum contraction whilst being verbally encouraged. Three trials were performed with each hand, with a 30 s rest period between trials to reduce fatigue.

#### Sensory testing

Digital fine-touch sensation was determined using the AsTex® (Australian Patent No.2008229741) screening tactile assessment tool [21]. The AsTex® is an acrylic board approximately 390 mm long and 100 mm wide, printed with parallel vertical ridges and grooves that logarithmically decline in width from 2.5 mm to 0.2 mm along its length [22]. Participants are requested to run their index finger across the grooves in the direction from widest to narrowest with their eyes shut, stopping when they can no longer perceive individual grooves. The point at which the participant stopped was recorded and the value transformed into a texture discrimination index (TDI) [22]. This was repeated for a total of three trials for each hand and the smallest TDI value for each participant was retained for statistical analysis.

### Statistical analysis

Demographic and other group characteristics were compared using non-parametric (categorical and non-normally distributed continuous data) and parametric tests (normally distributed continuous data). A chi square test was used to compare categorical group characteristics (group composition per locality, sex, handedness, musical history and video-gaming history). A general linear model univariate analysis was used to test for differences in normally distributed continuous demographic characteristics and test outcome variables. The Mann-Whitney U test was used for non-normally distributed data. Where indicated, sex was included in the statistical model as a covariate.

Age has been identified as a significant confounder for some psychomotor function tests [1, 16]. Thus, to ensure that within-group variability was not due to variation in age or experience levels, correlations were calculated (Pearson’s or Spearman’s Rho as appropriate) with the dependent test variables and age, and patient experience for the Experienced group.

Binomial logistic regression was used to directly compare significant outcomes from group comparisons in order to determine which tests contributed most to group categorisation. Group membership (Novice and Experienced) was the dependent variable with each of the test variables entered into the model as factors. Prior to establishing the regression model, the independent variables were checked for multicollinearity.

## Results

A total of 108 participants were recruited with 54 participants in each of the Novice and Experienced groups. A total of 85 participants were tested at UniSA and 23 at QUT.

### Group characteristics

Group characteristics can be found in Table 2.

**Table 2:** Comparison of Novice and Experienced group characteristics

	Novice (*n* = 54)	Experienced (*n* = 54)	Between group differences *p* values
Location			
UniSA	35 (64.8%)	50 (92.6%)	< 0.001**^
QUT	19 (35.2%)	4 (7.4%)	
Sex			
Men	21 (38.9%)	16 (29.6%)	0.311^
Women	33 (61.1%)	38 (70.4%)	
Age, (years) (SD)	22.5 (5.1)	30.7 (4.7)	< 0.001**††
Handedness			
Right	41 (75.9%)	46 (85.2%)	0.278^
Left	5 (9.3%)	5 (9.3%)	
Ambidextrous	8 (14.8%)	3 (5.6%)	
Height (cm) (SD)	171.1 (8.9)	171.7 (10.2)	0.771†
Musical History			
Yes	16 (29.6%)	27 (50%)	0.031*^
No	38 (70.4%)	27 (50%)	
Video Gaming History			
Yes	23 (42.6%)	16 (29.6%)	0.161^
No	31 (57.4%)	38 (70.4%)	

†† Mann-Whitney U test. † Univariate analysis. ^ Chi square analysis

* *p* < 0.05, ** *p* < 0.001

### Which dependent test outcomes significantly differentiate the experience between groups?

Only five of the eight tests (Tables 3 and 4): the GPT, the grip-lift task, P-MVC, G-MVC and AsTex® sensory test showed a significant difference between the two experience groups. The strength tests (P-MVC and G-MVC) were only significantly different between experience groups when sex was considered as a factor in the univariate analysis.

**Table 3:** Comparison of Novice and Experienced groups for tests of Tremor, VTT, FTT, P-MVC, GPT, G-MVC and the AsTex® sensory test

Test	Outcome	Novice (*n* = 54)	Experienced (*n* = 54)	*P*-value
		Mean (SD) or Median (IQR)		
Tremor	Maximum frequency (Hz)			
	Resting	2.60e^−8^ (3.89e^− 8^)	3.23e-8 (4.03e^− 8^)	0.509
	Self-paced	2.12e^−3^ (2.62e^− 3^)	1.58e-3 (1.82e^− 3^)	0.088
	Time of maximum (ms)			
	Resting	10.94 (1.56)	10.94 (4.30)	0.464
	Self-paced	9.57 (2.34)	9.38 (1.95)	0.829
	Mean Frequency (Hz)			
	Resting	1.37e^−8^ (1.50e^−8^)	1.31e^−8^ (1.37e^− 8^)	0.945
	Self-paced	1.16e^−3^ (1.53e^−3^)	8.71e^− 4^ (1.53e^− 3^)	0.143
Visuomotor tracking	Absolute Error (deg)			
	Epoch 1	4.19 (1.36)	3.80 (1.48)	0.151
	Epoch 2	3.78 (1.50)	3.93 (1.24)	0.701
	Epoch 3	3.66 (0.91)	3.70 (1.06)	0.626
	Maximum correlation value (ρ)			
	Epoch 1–3	0.30 (0.15)	0.33 (0.16)	0.312†
	Epoch 4–6	0.41 (0.20)	0.46 (0.20)	0.287†
	Epoch 7–12	0.55 (0.20)	0.54 (0.20)	0.850†
	Time lag (s)			
	Epoch 1–3	−0.30 (0.31)	−0.34 (0.25)	0.729
	Epoch 4–6	−0.25 (0.16)	−0.27 (0.13)	0.982
	Epoch 7–12	−0.25 (0.91)	−0.26 (0.09)	0.797
Finger-tapping	Maximum taps 10 s (n)			
	Dominant	62.25 (5.33)	62.02 (6.40)	0.839†
	Non-dominant	56.53 (5.90)	54.92 (5.53)	0.149†
	Coefficient variation (%)			
	Dominant	0.09 (0.09)	0.12 (0.12)	0.206
	Non-dominant	0.11 (0.12)	0.14 (0.12)	0.251
Grooved Pegboard	Time (s)			
	Dominant	53.77 (6.01)	51.06 (5.33)	0.015*†
	Non-dominant	60.39 (8.07)	56.94 (6.24)	0.014*†
AsTex® sensory test	TDI (mm)			
	Dominant	0.53 (0.41)	0.57 (0.45)	0.048*
	Non-dominant	0.52 (0.34)	0.54 (0.46)	0.728
Maximum Pinch Grip	Force (n)			
	Dominant			
	Male	81.47 (18.28)	91.17 (14.20)	0.039*
	Female	61.51 (17.05)	65.07 (12.73)	
	Non-dominant			
	Male	77.34 (16.42)	90.84 (14.80)	0.005*
	Female	58.26 (13.11)	60.28 (10.74)	
Grip Strength	Force (kg)			
	Dominant			
	Male	47.86 (8.39)	52.38 (9.04)	0.005*
	Female	29.70 (4.90)	32.79 (5.40)	
	Non-dominant			
	Male	44.19 (8.18)	48.56 (6.89)	0.009*
	Female	26.85 (5.48)	29.26 (5.33)	

* *p* < 0.05, ** *p* < 0.001

† indicates one-way ANOVA, means and standard deviations. †† Indicates a two-way ANOVA, means and standard deviations with Sex included as a factor

All other calculations utilised Mann-Whitney U non-parametric tests, medians and interquartile ratios for non-normally distributed data

**Table 4:** Comparison of Novice and Experienced groups for the Grip-Lift task variables

Test	Outcome	Novice (*n* = 54)	Experienced (*n* = 54)	*P*-value
		Mean (SD) or Median (IQR)		
Grip-Lift task	Preload duration (ms)			
	Dominant	97.5 (166.25)	286.25 (375.63)	< 0.001**
	Non-dominant	108.75 (88.13)	138.75 (359.69)	0.030*
	Minimum Load (N)			
	Dominant	−0.21 (0.17)	−0.19 (0.16)	0.252
	Non-dominant	−0.24 (0.20)	− 0.19 (0.16)	0.079
	Maximum grip force (N)			
	Dominant	5.20 (2.97)	6.02 (3.30)	0.167
	Non-dominant	5.24 (3.54)	6.75 (4.14)	0.033*
	GF:LF			
	Dominant	2.27 (1.65)	2.44 (1.34)	0.422
	Non-dominant	2.13 (1.27)	2.65 (1.72)	0.085
	Maximum correlation (ρ)			
	Dominant	0.74 (0.14)	0.71 (0.15)	0.012*
	Non-dominant	0.77 (0.08)	0.72 (0.11)	0.029*†
	Timeshift (ms)			
	Dominant	−8.75 (26.88)	−10.63 (40.94)	0.660
	Non-dominant	−5.00 (26.88)	−5.00 (36.88)	0.918
	Lift Duration (ms)			
	Dominant	292.50 (247.50)	433.75 (827.19)	< 0.001**
	Non-dominant	247.50 (208.75)	295.63 (413.75)	0.049*
	Average Grip (N)			
	Dominant	3.69 (2.61)	4.96 (2.83)	0.007*
	Non-dominant	3.82 (2.79)	4.94 (2.46)	0.012*
	SD Grip			
	Dominant	0.24 (0.11)	0.15 (0.07)	< 0.001**
	Non-dominant	0.23 (0.09)	0.15 (0.07)	< 0.001**
	Hold Ratio			
	Dominant	1.72 (1.28)	2.04 (1.05)	0.042*
	Non-dominant	1.61 (1.22)	1.99 (1.20)	0.038*

* *p* < 0.05, ** *p* < 0.001

† indicates one-way ANOVA, means and standard deviations

All other calculations utilised Mann-Whitney U non-parametric tests, medians and interquartile ratios for non-normally distributed data

For the Grip-lift task, seven dependent variables showed significant differences: PDn, Maximum cross-correlation, GFmax (Non-dominant hand only), GFavge, GFsd, LFDn and Hold Ratio (Table 4). In almost all of these measures, the Novice group outperformed the Experienced group. The only exception to this trend was on the GFsd variable where the Experienced group showed lower means and less within-group variation than the Novice group. The Novice group also outperformed the Experienced group on the AsTex sensory test. In contrast, the Experienced group performed better than the Novice group on the GPT and both strength tests P-MVC and G-MVC. The non-dominant hand on the P-MVC test showed a significant sex by experience group interaction, *p* = 0.037. Post-hoc comparisons of the sex sub-groups within the two experience groups suggest that it was the male sub-group driving the significant difference, however, neither of the sex sub-groups reached significance for the dominant hand.

### Which outcome measures best categorise participants appropriately into experience groups?

Current or previous ‘musical instrument’ use was included as a covariate as this was a significant demographic difference between the groups. After highly correlated (Table 5) and non-significant variables were progressively removed the final regression model contained six independent variables (Table 6). The full model containing predictors was significant X^2^ (6, *N* = 107) = 49.259, *p* <  0.001. The model as a whole explained between 36.9% (Cox and Snell R square) and 49.2% (Nagelkerke R Square) of the variance in group classification and correctly classified 79.4% of cases. The strongest predictor for classification into the Experienced group was the AsTex® dominant hand with a coefficient value (OR) of 4.73, indicating that participants who scored well (smaller value) were almost five times more likely to belong to the novice group when the other variables are held constant.

**Table 5:** Correlated predictor variables (for binary logistic regression)

	Dominant *(r)*	Non-dominant *(r)*
Average grip – Hold ratio	0.95**	0.96**
Average grip – Maximum Grip	0.93**	0.96**
Hold ratio – Maximum Grip	0.89**	0.92**

**p < 0.001

**Table 6:** Final model logistic regression calculated for predicted experience group classification

		B	S.E.	Wald	df	p	Odds Ratio	95% C.I.	
								Lower	Upper
Grip-Lift task									
Average Grip	Dominant	0.332	0.115	8.288	1	0.004*	1.394	1.112	1.747
	Dominant	−8.042	4.545	3.131	1	0.077	0.000	0.000	2.376
SD Grip	Non-Dominant	−11.062	4.946	5.002	1	0.025*	0.000	0.000	0.255
Load Duration	Dominant	0.001	0.001	5.210	1	0.022*	1.001	1.000	1.003
AsTex®									
TDI	Dominant	1.553	0.908	2.926	1	0.087	4.727	0.797	28.021
Musical Instrument		1.009	0.535	3.563	1	0.059	2.743	0.962	7.819
Constant		−0.772	1.193	0.419	1	0.518	0.462		

*p < 0.05, **p < 0.001

### Were there any within-group confounders for test variables based on actual level of experience in the experienced group?

Correlations were calculated to ensure that possible within-group variability of the Experienced group from ‘age’ and ‘experience’ were not detrimentally impacting on the dependent test variables. No significant correlations were present between estimated number of patients seen to date by the Experienced group participants and any of the dependent variables (Tables for this can be found in Additional file 2). Age only correlated with one dependent variable: the time at which the cross-correlation was at its maximum for epoch one to 3 seconds for the VTT (Lag 1–3, *r* = − 0.298, *p* = 0.030).

### Power analysis

Post-hoc power calculations showed sufficient power for the logistic regression analysis (α = 0.81). P-MVC was strongly powered (α = 0.86) and the GPT and G-MVC moderately powered (α = 0.70 and 0.77 respectively). The remaining tests did not reach adequate power.

### Post-hoc comparison of results

To further explain the results we also compared our results with normative values [16, 23–25], confirming that the Novice group performed better than would normally be expected.

## Discussion

Five of the eight tests used in the test battery were able to successfully discriminate dexterous ability of participants in the Novice and Experienced groups. These included the Grip-lift task, GPT, P-MVC, G-MVC and the AsTex® sensory discrimination test. These tests comprised a total of 11 significant dependent variables. A large proportion of group membership but less than 50% of the test variance could be explained by the test results.

Despite this, the results were different than anticipated, in particular with the Novice group outperforming the Experienced group on many of the Grip-lift task outcomes. For the force-related measurements (GFmax, GFavge, GFsd) a lower value suggests a superior performance, for temporal measures (PDn, LFDn) lower values indicate a superior performance and finally for ratio measures (Hold ratio) values closest to one suggest a superior performance. In healthy participants a scaling of Grip Force (GF) occurs to allow the object to be lifted (Lift Force (LF) to overcome gravity) without being too excessive so as to damage the object or hand, or give rise to increased muscle fatigue or affect the manipulation of that object [10]. The GF applied is dependent on four main factors: a predetermined motor plan based on prior experience, the weight of the object, the coefficient of friction of the object and the safety margin employed [9, 10, 26]. Thus, the poorer performance of the Experienced group may be the consequence of alterations in any of these factors. The small but significant difference on the AsTex® sensory test would support the theory this is related to afferent feedback. However, we would also expect that if this were the case then we should also see increased variability and therefore larger GFsd on the grip-lift task which also considers afferent feedback, which we did not. Instead, it may be that the experienced practitioners are less concerned with the issue of fatigue related to higher forces due to an increased endurance capacity, have chosen to forgo lower forces to enable greater stability and have developed strategies for maintaining the ability for object manipulation, or are more used to an increased safety margin as a result of regular glove usage [27, 28], practice requirements or similar.

It has also been consistently found that older adults use a higher level and fluctuation in grip force during movement and static hold [29–32], suggesting age may potentially play a part in explaining the findings. These age-related mechanisms are reported to be influential in populations 50 years old and above [30], so it seems unlikely that they would be the primary reason to explain the outcomes in this study, but as this is likely to be a progressive change, the influence of earlier changes should not be ignored.

Gilles and Wing [31] found that grip-lift trials of less than 4.7 ms^− 2^ vertical acceleration were associated with quite variable GF measures and subsequently excluded these trials from their analysis. We were unable to measure the velocity of the lift accurately; however, there was a significantly slower LFdn mean for the Experienced group, which could have influenced measures. Kinoshita and Francis [32] noted that for their older cohort this was due to a prolonged PDn. Preload duration was similarly prolonged for the Experienced group, particularly for the dominant hand, in this study.

For all of the significant variables from the remaining tests (P-MVC, G-MVC, GPT) except the AsTex® sensory test, the Experienced group outperformed the Novice group. The superior haptic threshold performance by the Experienced group as determined by the GPT was consistent with findings by Mueller et al. [33] who found improved haptic performance in physiotherapists and advanced manual therapists compared with a control group. They also found a decreased haptic threshold in Physiotherapy students compared to control, similar to our findings. Mueller and colleagues [33] found a large variance in individual performance within the groups, which they attributed to individual differences in everyday tactile perceptual learning and reliance on touch [34]. Interestingly, Mueller et al. [33] did not find a significant difference in tactile acuity between therapists and control groups measured with grating domes. The question remains as to whether improved haptic threshold translates to improved manual dexterity.

The conflicting results between the grip-lift task, AsTex® sensory test and other tests could relate to the psychometric properties of the specific test and the skill-set required for podiatric tasks. Interestingly, the GPT has been found to correlate with grip strength, pinch grip strength and pinch steadiness tests [1, 35]. This is supported by the results in this study.

Considering the psychometric properties of the tests at face value, these results suggest that the Novice group performed better on the tests which require high levels of sensory elements and subsequent adaptation, whereas the Experienced group performed better on tests which required the combination of speed, strength and precision. This suggests the possibility that experienced practitioners rely less on sensory feedback.

Another possibility is that the tests used were too generalised or did not contain the required combination of elements to represent the associated task-related dexterity. This has been discussed in the literature whereby various medical and surgical professions such as those using endoscopy have recently found success using virtual reality (VR) trainers to train and evaluate manual clinical skills [36–39].

## Conclusion

This study showed that a variety of elements contributed to the differentiation of novice students and experienced podiatrists. The outcomes representing these elements could predict approximately 79% of the group membership overall. The greatest contributor to group membership was the test of sensory perception for the dominant hand (AsTex®). The Experienced group displayed superior performance in strength and speed on the relevant GDTs; however, the Novice group showed superior coordination on the Grip-lift task and the dominant hand on the AsTex® Sensory test. The strength tests were affected by the sex of participants and therefore test results need to be interpreted in relation to an individual’s sex. Thus, future dexterity testing on this population should use the GPT, Grip-lift task, Grip Strength test and Pinch Grip Strength test. However, we would only recommend these tests be used to evaluate dexterous change relating to an intervention, rather than as a threshold.

## Additional files

Additional file 1: Grip-Lift task operational definitions and outcomes of interest. This provides a more in-depth of the definitions used for each of the outcomes of interest analysed from the data for the Grip-Lift task. (DOCX 125 kb)

Additional file 2: Correlation tables for within-group age and experience variables. This provides the tables outlining the statistical analysis for correlations between within-group characteristics of age and number of patients with the outcomes of interest for the Experienced group. (DOCX 23 kb)

## Abbreviations

dGF/dt: Rate of Change of Grip Force over time
dLF/dt: Rate of change of Lift Force over time
EHI: Edinburgh Handedness Inventory
FTT: Finger Tapping Test
GDT’s: Generalised dexterity tests
GF: Grip Force
GF:LF: Grip Force to Lift Force Ration
GFavg: Average Grip Force
GFmax: Maximum Grip Force
GFsd: Standard Deviation of Grip Force
G-MVC: Grip Strength or Grip Maximum Voluntary Contraction
GPT: Grooved Pegboard Test
IQR: Interquartile Range
LF: Lift Force
LFDn: Lift Force Duration
LFmin: Minimum Load
PDn: Preload Duration
P-MVC: Pinch Grip or Pinch Maximum Voluntary Contraction
QUT: Queensland University of Technology
SD: Standard Deviation
TDI: Texture Discrimination Index
UniSA: University of South Australia
VR: Virtual Reality
VTT: Visuomotor Tracking Task
