# Supplementary material for: Measuring dexterity in the podiatrist population: a cross-sectional comparison of novice students and experienced podiatrists
Source: BMC Med Educ. 2018 Aug 2;18:181. doi: 10.1186/s12909-018-1276-1 (PMC6090916; doi:10.1186/s12909-018-1276-1)
Supplement: Supplementary file 1 — Grip-Lift task operational definitions and outcomes of interest. This provides a more in-depth of the definitions used for each of the outcomes of interest analysed from the data for the Grip-Lift task. (DOCX 125 kb) [file 12909_2018_1276_MOESM1_ESM.docx]

## Grip-Lift task operational definitions and outcomes of interest

Operational definitions

- Pre-lift phase comprises the 0.5 s prior to grip onset as determined below (Figure 3 – T_0_-T_1_) and is used to calculate the baseline values for GF and LF channels
- Grip onset occurs at the last point when the grip lift signal derivative (slope) is at zero prior to the signal reaching 30% of maximum force (Figure 3 – T_1_)
- Lift onset occurs when the lift force becomes positive prior to the signal reaching 50% of maximum force (Figure 3 – T_2_)
- Lift phase incorporates up to 2.5 s prior to maximum grip force (Figure 3 – T_0_-T_3_)
- Hold phase incorporates a 1.5 s period starting 1 s after maximum grip force is reached (Figure 3 – T_4_-T_5_)

Outcomes of interest

- **Preload duration (PDn)** (ms) (Figure 3: T_1_-T_2_): time between onset of Grip Force (GF) and onset of positive Lift Force (LF). This may be preceded by a negative LF if the manipulandum is pushed into the supporting surface. A longer period corresponds with a clumsy lift strategy.
- **Minimum load (LFmin)** (N): maximum downwards force applied during PDn if the object was pushed into the surface as part of the lift phase. The greater the force the poorer the technique when attempting to grip and lift the manipulandum.
- **Maximum grip force (GFmax)** (N): peak GF reached during the lift phase. Optimum GF is the lowest force applied without slippage occurring and is made up of the force required to overcome LF plus a margin of error. Therefore, the higher the GF applied the greater the margin of error, and considered a poorer performance.
- **Grip force to Lift force ratio (GF:LF)**: ratio of GF to LF at GFmax during the lift phase. A low ratio suggests a better scaling of required force and therefore a better grip-lift strategy.
- **Maximal cross-correlation:** the maximum correlation coefficient obtained when the rate of change of GF over time (dGF/dt) and rate of change of LF over time (dLF/dt) were cross-correlated using time-shifts of 2.5 ms [14]. A cross-correlation value of one indicates a perfect response of the participant’s grip force regulation synchronous to the increase in lift force.
- **Time-shift** (ms): the incremental shift required for maximum cross-correlation of dGF/dt and dLF/dt. This is calculated using 2.5 ms increments as outlined above until maximum cross-correlation is obtained. The time-shift is an indicator of whether the grip was primarily anticipatory or reactive. A positive time-shift indicates an anticipatory strategy and conversely a negative time-shift indicates a reactive strategy.
- **Lift Duration (LFDn)** (Figure 3 – T_2_-T_3_): The time from which LF onset occurs until the force reaches within 98% of maximum LF. Previous studies have found poorer strategies to be associated with a longer lifting duration [14].
- **Average grip force (GFavg)** (N): The average GF during the hold phase. A higher average force is indicative of poor scaling of force relative to the weight and therefore poorer lift strategy.
- **Standard Deviation of Grip Force (GFsd)**: The standard deviation of the GF during hold phase, i.e. how much the GF varies whilst trying to maintain the manipulandum at the required height [40]. Less change in force over this time results in lower standard deviations, indicating better control.
- **Hold ratio**: The average ratio of GF to LF as the object is held stationary during the hold phase. If the participant uses greater force to hold the manipulandum stationary, suggesting a less efficient technique, then the ratio increases.


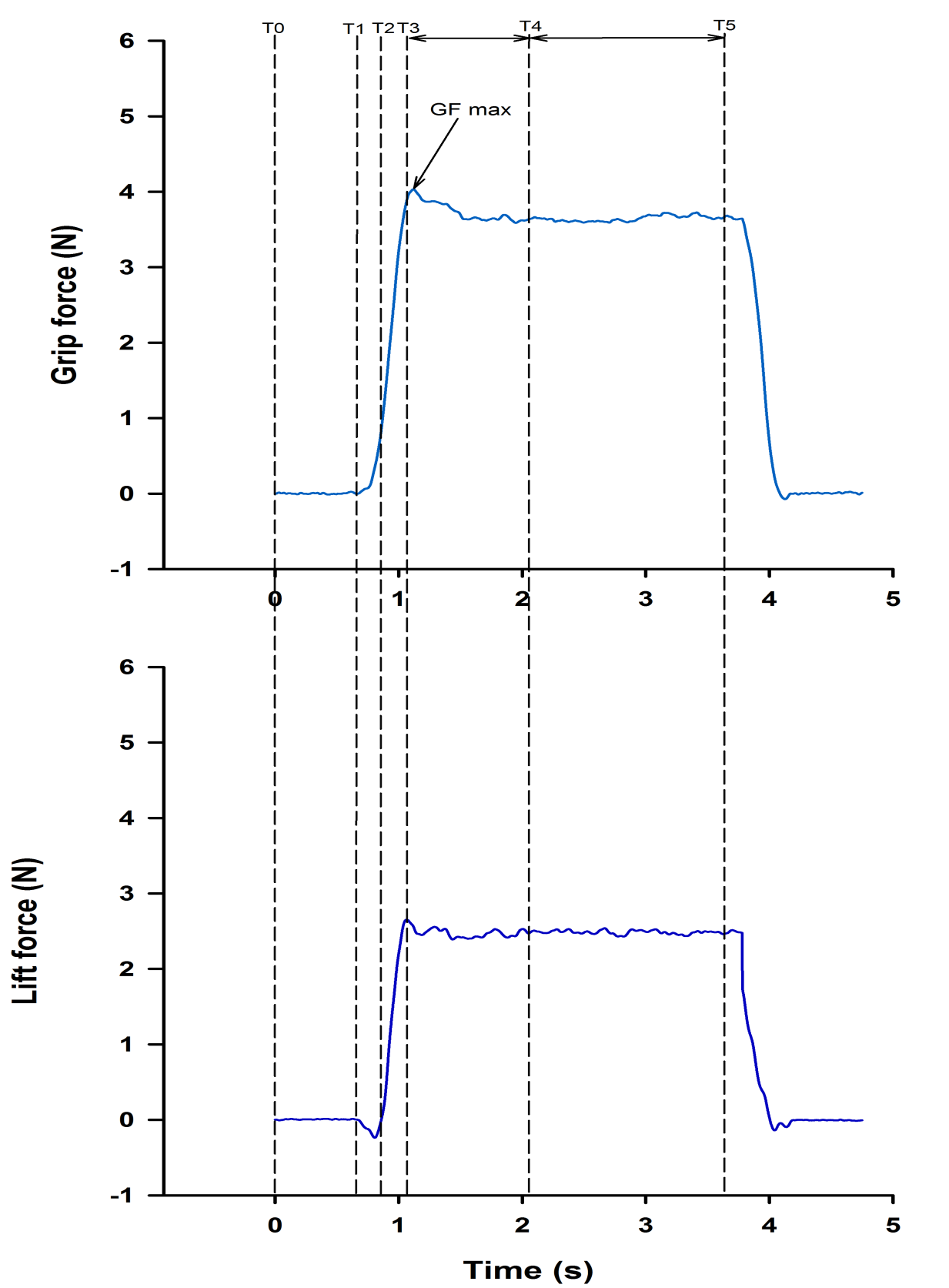


- T_0_ – T_1_ = Pre-lift Phase (Baseline calculation)
- T_1_ – T_2_ = Preload Duration (PDn)
- T_1_ – T_3_ = Lift Duration (LFDn)
- T_0_ – T_3_ = Lift Phase
- T_4_ – T_5_ = Hold Phase

Figure 3: Actual example of a lift with indicative time points used for variable calculation (based on a diagram by Duque et al., [14].
