# Supplementary material for: Measuring dexterity in the podiatrist population: a cross-sectional comparison of novice students and experienced podiatrists
Source: BMC Med Educ. 2018 Aug 2;18:181. doi: 10.1186/s12909-018-1276-1 (PMC6090916; doi:10.1186/s12909-018-1276-1)
Supplement: Supplementary file 2 — Correlation tables for within-group age and experience variables. This provides the tables outlining the statistical analysis for correlations between within-group characteristics of age and number of patients with the outcomes of interest for the Experienced group. (DOCX 23 kb) [file 12909_2018_1276_MOESM2_ESM.docx]

## Correlation tables for within-group age and experience variables

Table S7 Correlations of Estimated patients and age with VTT, P-MVC, FTT, GPT, G-MVC and AsTEX® sensory test variables for the Experienced Group

|  |  | **Estimated patients** | | **Age** | |
| --- | --- | --- | --- | --- | --- |
|  |  | *Correlation* | *P-value* | *Correlation* | *P-value* |
| **Estimated patients** | |  |  | **0.62** | ***<0.001***** |
| **VTT**  Error 1†  Error 2†  Error 3†  Max r 1-3  Max r 4-6  Max r 7-12  lag 1-3†  lag 4-6†  lag 7-12†  lag 13-18† |  |  |  |  |  |
|  |  | -0.06 | 0.67 | -0.17 | 0.23 |
|  |  | 0.04 | 0.79 | -0.07 | 0.60 |
|  |  | 0.03 | 0.86 | -0.16 | 0.26 |
|  |  | -0.02 | 0.90 | 0.13 | 0.34 |
|  |  | -0.03 | 0.84 | 0.04 | 0.78 |
|  |  | -0.07 | 0.63 | 0.13 | 0.36 |
|  |  | -0.10 | 0.47 | **-0.30** | ***0.03**** |
|  |  | -0.12 | 0.42 | -0.02 | 0.92 |
|  |  | 0.01 | 0.96 | 0.08 | 0.56 |
|  |  | 0.04 | 0.77 | 0.01 | 0.95 |
| **P-MVC** |  |  |  |  |  |
|  | Dominant | -0.05 | 0.72 | 0.05 | 0.74 |
|  | Non-Dominant | 0.06 | 0.66 | 0.08 | 0.57 |
| **FTT**  No. of taps  CV |  |  |  |  |  |
|  | Dominant | 0.05 | 0.73 | 0.05 | 0.70 |
|  | Non-dominant | 0.08 | 0.57 | 0.14 | 0.31 |
|  | Dominant† | 0.02 | 0.91 | 0.16 | 0.26 |
|  | Non-dominant† | 0.03 | 0.85 | -0.13 | 0.37 |
| **GPT**  Time to completion |  |  |  |  |  |
|  | Dominant | 0.15 | 0.28 | 0.04 | 0.75 |
|  | Non-dominant | -0.15 | 0.30 | -0.09 | 0.50 |
| **G-MVC** |  |  |  |  |  |
|  | Dominant | 0.13 | 0.34 | 0.05 | 0.71 |
|  | Non-dominant | 0.12 | 0.41 | 0.07 | 0.62 |
| **AsTex^®^**  **sensory test** |  |  |  |  |  |
|  | Dominant† | 0.08 | 0.56 | 0.00 | 0.97 |
|  | Non-dominant† | 0.02 | 0.91 | 0.18 | 0.19 |

†Indicates Spearman’s Rho correlation coefficient, all other calculations are Pearson’s correlation. r = Correlation. CV = Coefficient of variation

* p < 0.05, ** p < 0.001

Table S8 Correlations of Estimated patients and age with Grip-Lift test variables for the Experienced Group

|  |  | **Estimated patients** | | **Age** | |
| --- | --- | --- | --- | --- | --- |
| **Grip-Lift Task** |  | *Correlation* | *P-value* | *Correlation* | *P-value* |
| **PDn**  **GFmin**  **GFmax**  **GF:LF**  **Maximum Correlation**  **Timeshift**  **LFDn**  **GFavge**  **GFsd**  **Hold Ratio** | Dominant† | 0.10 | 0.49 | 0.20 | 0.15 |
|  | Non-dominant† | -0.02 | 0.89 | -0.10 | 0.47 |
|  | Dominant† | 0.03 | 0.83 | 0.11 | 0.43 |
|  | Non-dominant† | 0.02 | 0.88 | 0.08 | 0.59 |
|  | Dominant† | -0.02 | 0.90 | -0.13 | 0.35 |
|  | Non-dominant† | -0.08 | 0.58 | 0.04 | 0.78 |
|  | Dominant† | -0.01 | 0.92 | -0.15 | 0.27 |
|  | Non-dominant† | -0.08 | 0.59 | 0.06 | 0.69 |
|  | Dominant† | -0.10 | 0.49 | -0.05 | 0.72 |
|  | Non-dominant† | -0.05 | 0.75 | -0.05 | 0.74 |
|  | Dominant† | 0.13 | 0.35 | -0.03 | 0.83 |
|  | Non-dominant† | -0.21 | 0.14 | -0.05 | 0.71 |
|  | Dominant† | -0.09 | 0.50 | -0.04 | 0.80 |
|  | Non-dominant† | 0.14 | 0.31 | 0.16 | 0.25 |
|  | Dominant† | -0.03 | 0.82 | -0.07 | 0.61 |
|  | Non-dominant† | -0.06 | 0.68 | 0.07 | 0.60 |
|  | Dominant† | 0.08 | 0.59 | 0.00 | 0.98 |
|  | Non-dominant† | -0.17 | 0.23 | -0.13 | 0.37 |
|  | Dominant† | -0.01 | 0.97 | -0.08 | 0.57 |
|  | Non-dominant† | -0.03 | 0.82 | 0.08 | 0.58 |

†Indicates Spearman’s Rho correlation coefficient, all other calculations are Pearson’s correlation
